# Epidemics of Vector-borne Diseases Observed in Infectious Disease Surveillance in Japan, 2000-2005

**DOI:** 10.2188/jea.17.S48

**Published:** 2008-01-30

**Authors:** Shuji Hashimoto, Miyuki Kawado, Yoshitaka Murakami, Michiko Izumida, Akiko Ohta, Yuki Tada, Mika Shigematsu, Yoshinori Yasui, Kiyosu Taniguchi, Masaki Nagai

**Affiliations:** 1 Department of Hygiene, Fujita Health University School of Medicine.; 2 Department of Health Science, Shiga University of Medical Science.; 3 Department of Public Health, Saitama Medical University Faculty of Medicine.; 4 Infectious Disease Surveillance Center, National Institute of Infectious Diseases.

**Keywords:** Disease Vectors, Disease Notification, Dengue, Japanese Spotted Fever, Scrub Typhus

## Abstract

**BACKGROUND:**

Observing the epidemics of vector-borne diseases is important. One or more cases of 6 vector-borne diseases were reported to the National Epidemiological Surveillance of Infectious Diseases in Japan in 2000-2005.

**METHODS:**

The reports of those cases were available. The incidence was observed by region of acquired infection, prefecture reporting, and week and year of diagnosis.

**RESULTS:**

The incidence rate per year per 1,000,000 population was 0.36 for dengue fever, 0.04 for Japanese encephalitis, 0.38 for Japanese spotted fever, 0.08 for Lyme disease, 0.74 for malaria, and 3.50 for scrub typhus. There were no cases of dengue fever or malaria derived from domestic infections. The yearly incidence rate increased for dengue fever and Japanese spotted fever, and declined for malaria and scrub typhus. The proportion of cases reported in Tokyo was 44% for dengue fever and 37% for malaria. The number of prefectures reporting one or more cases of Japanese spotted fever increased in western Japan. The cases of scrub typhus increased in autumn-winter in prefectures of eastern Japan, and increased both in autumn-winter and spring in western prefectures.

**CONCLUSIONS:**

The study reveals the epidemiologic features of both temporal and geographic distributions of cases of 6 vector-borne diseases in Japan, 2000-2005.

Vector-borne diseases, such as dengue fever and malaria, have a major impact on public health all over the world.^1^ There are disease-specific characteristics in geographic distribution, temporal trends and seasonality of such cases because their transmission is dependent on the spread and density of appropriate vectors (mosquito, tick, etc).^2^ In many countries, the surveillance of various vector-borne diseases has been conducted with the aim of detecting, controlling and preventing their epidemics.^3^^-^^6^ The epidemiologic characteristics of those diseases have been described from the surveillance data.^3^^,^^4^

In Japan, the National Epidemiological Surveillance of Infectious Diseases (NESID) has targeted specific vector-borne diseases.^6^^,^^7^ Those with one or more cases reported in 2000-2005 include 6 diseases: dengue fever, Japanese encephalitis, Japanese spotted fever, Lyme disease, malaria and scrub typhus (tsutsugamushi disease).^7^ The epidemiologic features of these diseases have been described,^8^^-^^12^ but have not be sufficiently evaluated from the viewpoint of geographic and temporal clustering.

In the present study, the geographic and temporal distributions of cases of the above 6 vector-borne diseases were analyzed from the NESID data in Japan, 2000-2005.

## METHODS

### Surveillance of infectious diseases in Japan

NESID in Japan has been described elsewhere.^6^^,^^7^^,^^13^ Any physician who has diagnosed a notifiable disease must report the patient information to local public health center. Notification by public health centers to the local government (prefecture) and the Ministry of Health, Labour and Welfare of Japan is made through an on-line computer network.

A total of 11 vector-borne diseases are notifiable: the above 6 as well as relapsing fever, yellow fever, Crimean-Congo hemorrhagic fever, epidemic typhus, and plague.^6^^,^^7^ The information reported includes sex, age, date of diagnosis, and region where infection was acquired.

### Surveillance data and method of analysis

The reports involving the above 6 vector-borne diseases diagnosed in 2000-2005 to the NESID in Japan were available. The data we used were the week and year of diagnosis, prefecture reporting, and region of acquired infection (Japan, others, and unknown).

The incidence of 6 vector-borne diseases was observed by region acquired, prefecture reporting, and week and year of diagnosis. The incidence rate per population by prefecture reporting was calculated using the 2000 census population data, and was compared with that nationwide. The exact test under the assumption that the number of cases follows a Poisson distribution was used for the comparison.

## RESULTS

Table 1 shows the incidence of vector-borne diseases in 2000-2005. The total incidence and the incidence rate per year per 1,000,000 population were, respectively, 275 and 0.36 for dengue fever, 33 and 0.04 for Japanese encephalitis, 294 and 0.38 for Japanese spotted fever, 60 and 0.08 for Lyme disease, 566 and 0.74 for malaria, and 2,680 and 0.35 for scrub typhus. The yearly incidence rate rose for dengue fever and Japanese spotted fever, and declined for malaria and scrub typhus.

**Table 1.** Incidence of vector-borne diseases, Japan, 2000-2005.

Vector-borne diseases	Year						Total

	2000	2001	2002	2003	2004	2005	
Dengue fever	18	50	52	32	49	74	275 (0.36)
Japanese encephalitis	7	5	8	1	5	7	33 (0.04)
Japanese spotted fever	38	40	36	52	66	62	294 (0.38)
Lyme disease	12	15	15	5	5	8	60 (0.08)
Malaria	154	109	83	78	75	67	566 (0.74)
Scrub typhus	791	491	338	402	313	345	2,680 (3.50)

Incidence rates per year per 1,000,000 population in parentheses.

Table 2 shows the incidence of vector-borne diseases by region of acquired infection in 2000-2005. While nobody acquired the infection of dengue fever or malaria in Japan, the proportion of infection for the other 4 diseases was 86.7-100.0%.

**Table 2.** Incidence of vector-borne diseases by region of acquired infection, Japan, 2000-2005.

Vector-borne diseases	Region of acquired infection					Total

	Japan		Others		Unknown	
Dengue fever	0	(0.0)	275	(100.0)	0 (0.0)	275 (100)
Japanese encephalitis	33	(100.0)	0	(0.0)	0 (0.0)	33 (100)
Japanese spotted fever	294	(100.0)	0	(0.0)	0 (0.0)	294 (100)
Lyme disease	52	(86.7)	8	(13.3)	0 (0.0)	60 (100)
Malaria	0	(0.0)	557	(98.4)	9 (1.6)	566 (100)
Scrub typhus	2,669	(99.6)	6	(0.2)	5 (0.2)	2,680 (100)

Percentages in parentheses.

**Figure 1.**
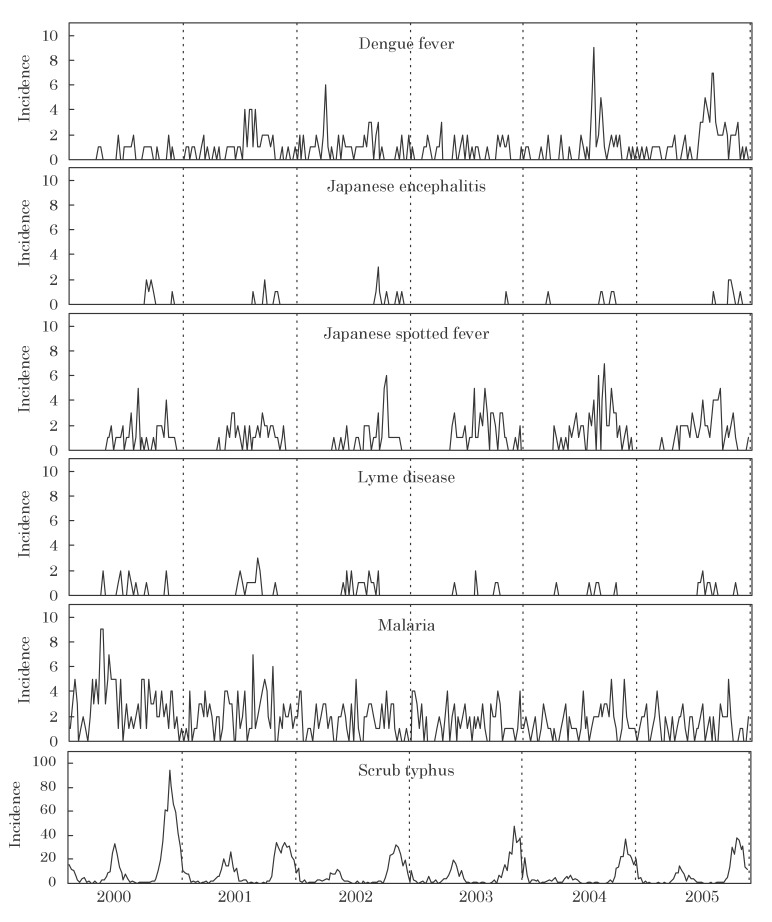
Incidence of vector-borne diseases by week and year of diagnosis, Japan, 2000-2005.

Figure 1 shows the incidence of vector-borne diseases by week and year of diagnosis in 2000-2005. Some seasonal patterns of incidence were observed for Japanese spotted fever and scrub typhus, but none for dengue fever and malaria.

Table 3 shows the incidence of vector-borne diseases by prefecture reporting in 2000-2005. The dengue fever cases reported in Tokyo were 43.6% of the national total. The incidence of Japanese encephalitis by prefecture was 4 cases or less. The incidence rate ratio of Japanese spotted fever compared with the national rate was over 3 in several prefectures of western Japan: Wakayama, Shimane, Tokushima, Ehime, Kochi, Miyazaki, and Kagoshima. Those ratios being higher than one were statistically significant. The cases of Lyme disease reported in Hokkaido, Japan's northernmost island, were 45.0% of the national total, while malaria cases reported in Tokyo were 37.2%. The incidence rate ratio of scrub typhus was over 3 in several prefectures in eastern and western Japan: Akita, Fukushima, Oita, Miyazaki, and Kagoshima. Those ratios being higher than one were statistically significant.

**Table 3.** Incidence of vector-borne diseases by prefecture reporting, Japan, 2000-2005.

Prefecture reporting	Dengue fever		Japanese encephalitis		Japanese spotted fever		Lyme disease		Malaria		Scrub typhus	
Hokkaido	4	(0.33)	0	(0.00)	0	(0.00)	27	(10.09) *	14	(0.55)	0	(0.00)
Aomori	0	(0.00)	0	(0.00)	0	(0.00)	1	(1.44)	1	(0.15)	78	(2.51) *
Iwate	0	(0.00)	0	(0.00)	0	(0.00)	0	(0.00)	0	(0.00)	41	(1.38)
Miyagi	6	(1.17)	0	(0.00)	0	(0.00)	1	(0.89)	6	(0.57)	30	(0.60)
Akita	1	(0.39)	0	(0.00)	0	(0.00)	0	(0.00)	0	(0.00)	160	(6.42) *
Yamagata	1	(0.37)	0	(0.00)	0	(0.00)	1	(1.71)	2	(0.36)	55	(2.11) *
Fukushima	1	(0.22)	0	(0.00)	0	(0.00)	2	(2.00)	3	(0.32)	220	(4.92) *
Ibaraki	3	(0.46)	0	(0.00)	0	(0.00)	0	(0.00)	10	(0.75)	13	(0.21)
Tochigi	3	(0.69)	0	(0.00)	0	(0.00)	0	(0.00)	12	(1.34)	17	(0.40)
Gunma	3	(0.68)	0	(0.00)	0	(0.00)	0	(0.00)	0	(0.00)	67	(1.57) *

Saitama	8	(0.53)	0	(0.00)	0	(0.00)	1	(0.30)	20	(0.64)	5	(0.03)
Chiba	23	(1.78) *	0	(0.00)	13	(0.94)	0	(0.00)	18	(0.68)	172	(1.37) *
Tokyo	120	(4.58) *	0	(0.00)	0	(0.00)	9	(1.57)	211	(3.91) *	50	(0.20)
Kanagawa	29	(1.57) *	0	(0.00)	0	(0.00)	4	(0.99)	65	(1.71) *	110	(0.61)
Niigata	0	(0.00)	0	(0.00)	0	(0.00)	1	(0.86)	7	(0.64)	113	(2.17) *
Toyama	3	(1.24)	0	(0.00)	0	(0.00)	1	(1.89)	1	(0.20)	16	(0.68)
Ishikawa	1	(0.39)	1	(3.26)	0	(0.00)	0	(0.00)	1	(0.19)	6	(0.24)
Fukui	1	(0.56)	0	(0.00)	1	(0.52)	0	(0.00)	3	(0.81)	3	(0.17)
Yamanashi	0	(0.00)	0	(0.00)	0	(0.00)	0	(0.00)	0	(0.00)	4	(0.21)
Nagano	3	(0.62)	0	(0.00)	1	(0.19)	2	(1.91)	1	(0.10)	43	(0.92)

Gifu	1	(0.22)	0	(0.00)	0	(0.00)	1	(1.01)	2	(0.21)	107	(2.41) *
Shizuoka	5	(0.61)	1	(1.02)	1	(0.11)	0	(0.00)	9	(0.54)	58	(0.73)
Aichi	13	(0.85)	0	(0.00)	0	(0.00)	1	(0.30)	30	(0.95)	19	(0.13)
Mie	2	(0.50)	1	(2.07)	2	(0.47)	0	(0.00)	5	(0.60)	19	(0.49)
Shiga	4	(1.37)	0	(0.00)	0	(0.00)	0	(0.00)	2	(0.33)	3	(0.11)
Kyoto	4	(0.70)	0	(0.00)	0	(0.00)	0	(0.00)	13	(1.11)	3	(0.05)
Osaka	16	(0.84)	1	(0.44)	1	(0.05)	1	(0.24)	49	(1.25)	5	(0.03)
Hyogo	5	(0.42)	0	(0.00)	18	(1.40)	2	(0.76)	18	(0.73)	31	(0.26)
Nara	5	(1.61)	1	(2.68)	0	(0.00)	0	(0.00)	2	(0.31)	0	(0.00)
Wakayama	1	(0.43)	1	(3.62)	13	(5.28) *	0	(0.00)	2	(0.42)	33	(1.47)

Tottori	0	(0.00)	1	(6.29)	1	(0.71)	1	(3.46)	3	(1.10)	19	(1.47)
Shimane	0	(0.00)	2	(10.14)	66	(37.55) *	0	(0.00)	4	(1.18)	35	(2.18) *
Okayama	0	(0.00)	4	(7.90) *	0	(0.00)	0	(0.00)	3	(0.35)	16	(0.39)
Hiroshima	1	(0.16)	4	(5.36) *	2	(0.30)	1	(0.74)	7	(0.55)	90	(1.48) *
Yamaguchi	0	(0.00)	2	(5.06)	0	(0.00)	1	(1.39)	2	(0.30)	5	(0.16)
Tokushima	1	(0.56)	0	(0.00)	21	(11.06) *	0	(0.00)	1	(0.27)	11	(0.64)
Kagawa	1	(0.45)	0	(0.00)	0	(0.00)	0	(0.00)	1	(0.22)	1	(0.05)
Ehime	0	(0.00)	1	(2.59)	12	(3.48) *	0	(0.00)	8	(1.21)	1	(0.03)
Kochi	0	(0.00)	2	(9.49)	61	(32.49) *	0	(0.00)	0	(0.00)	32	(1.87) *
Fukuoka	3	(0.28)	3	(2.30)	1	(0.09)	1	(0.42)	12	(0.54)	15	(0.14)

Saga	0	(0.00)	3	(13.21) *	0	(0.00)	0	(0.00)	0	(0.00)	21	(1.14)
Nagasaki	1	(0.31)	2	(5.10)	0	(0.00)	0	(0.00)	3	(0.45)	68	(2.13) *
Kumamoto	1	(0.25)	2	(4.15)	1	(0.23)	0	(0.00)	3	(0.36)	61	(1.56) *
Oita	0	(0.00)	1	(3.16)	1	(0.35)	0	(0.00)	0	(0.00)	117	(4.55) *
Miyazaki	0	(0.00)	0	(0.00)	17	(6.30) *	0	(0.00)	2	(0.38)	234	(9.51) *
Kagoshima	0	(0.00)	0	(0.00)	61	(14.81) *	1	(1.19)	4	(0.50)	472	(12.57) *
Okinawa	1	(0.35)	0	(0.00)	0	(0.00)	0	(0.00)	6	(1.02)	1	(0.04)

Ratios of incidence rate to that in whole of Japan in parentheses.

* p<0.01 by exact test for comparing with incidence rate in the whole of Japan.

**Figure 2.**
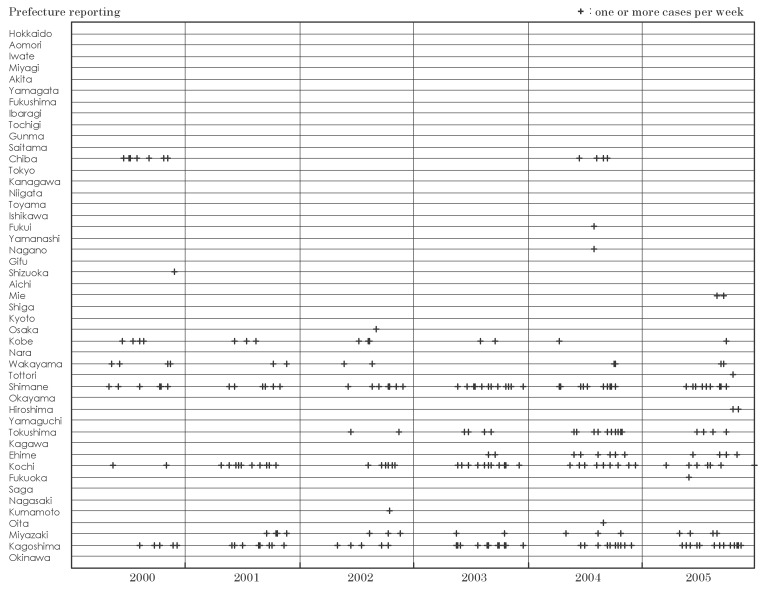
Distribution of cases of Japanese spotted fever by prefecture reporting, week and year of diagnosis, Japan, 2000-2005.

**Figure 3.**
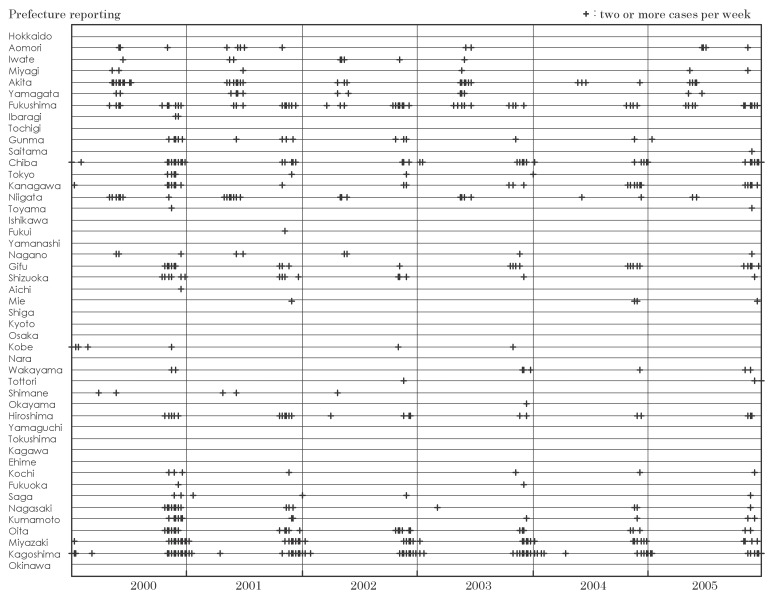
Distribution of cases of scrub typhus by prefecture reporting, week and year of diagnosis, Japan, 2000-2005.

Figures 2 and 3 show the distribution of cases of Japanese spotted fever and scrub typhus by prefecture reporting, and week and year of diagnosis in 2000-2005, respectively. Distributions of other diseases were not shown since the cases of Japanese encephalitis by prefecture were too few and the proportion of cases of dengue fever, Lyme diseases and malaria reported only in one prefecture was high, as shown in Table 3.

Cases of Japanese spotted fever (one or more cases in a week denoted as '+' in Figure 2) in Shimane, Kochi and Kagoshima were reported annually in 2000-2005. The reporting of cases in Miyazaki, Tokushima and Ehime started from 2001, 2002 and 2003, respectively. The number of prefectures reporting one or more cases increased from 6-9 in 2000-2003 to 12 in 2004 and 2005. Cases of scrub typhus (two or more cases in a week denoted as '+' in Figure 3) were reported in most prefectures in 2000-2005. Reported cases in several prefectures in eastern Japan increased in autumn-winter, while those in the west increased in both autumn-winter and spring.

## DISCUSSION

Dengue fever and malaria are transmitted by the bite of infected mosquitoes.^2^ No cases of anyone in Japan acquiring these infections were reported in 2000-2005, suggesting that domestic infections were highly unlikely to occur during this period, and that most cases encountered were almost certainly acquired while traveling in endemic areas and developing symptoms after returning home.^8^^,^^11^ Most cases of dengue fever and malaria observed in this study were reported in Tokyo, a finding that reflects the many travelers returning home through airports and seaports. The incidence of dengue fever increased in this period, while that of malaria decreased. The rise in dengue fever might be associated with the increased opportunities for infection due to the spread of endemic areas worldwide and with the rising coverage of diagnosis due to the enhanced awareness of physicians to this disease.^8^^,^^14^ The decrease in malaria might be attributable to more widespread prevention measures using several methods, such as chemoprophylaxis.^11^^,^^15^

Japanese encephalitis is a mosquito-borne disease, with many cases occurring in Japan during the 1950s, but falling dramatically to several dozen by the 1980s.^2^^,^^9^ In this study, it was observed that the incidence rate was stable at under 0.1 per year per 1,000,000 population in 2000-2005. The leading reason for such dramatic improvement was that most children acquired protective immunity to the Japanese encephalitis virus through an increase in vaccination programs.^9^^,^^16^

Lyme disease is a tick-borne infection endemic to the United States, and eastern and central Europe.^2^^,^^17^ The tick mainly transmitting Lyme disease infection in Japan is most prevalent in Japan's northernmost island of Hokkaido and in the mountains of central and northern Japan.^18^ The high proportion of cases reported in Hokkaido would be associated with the distribution pattern of those ticks.

Japanese spotted fever as a tick-borne disease was first reported in Japan in 1984.^10^ In this study, we observed the spread of temporal and geographic distributions of cases in 2000-2005. Our results were similar to those reported in previous studies.^10^^,^^19^ One reason for the spread of cases might be that the distribution of infected vector ticks spread during this period.

Scrub typhus is transmitted by the attaching of infective trombiculid mites, and has been endemic all over Japan except for a few prefectures.^10^^,^^12^ The incidence of cases was observed to fall from 791 in 2000 to 345 in 2005. Though the reason for the decrease is unknown, some interesting seasonal and geographic patterns of infections were reported in previous studies.^10^^,^^20^ Such pattern have been related to the activities of two different species of trombiculid mites, insofar as the high incidences in autumn-winter in many areas were mainly due to one species of mite, while those in spring in western Japan were mainly due to the other.

In conclusion, although there were some limitations and problems in the present study, based as it was only on reports to the NESID, some meaningful epidemiologic features in the temporal and geographic distributions of cases of 6 vector-borne diseases in Japan, 2000-2005, were revealed.
